# Epidural Anesthesia for Cesarean Section in a Pregnant Woman With Acute Pulmonary Edema: A Case Report

**DOI:** 10.7759/cureus.43994

**Published:** 2023-08-23

**Authors:** Barbara Alves, Filipa Cunha, Cláudia Pereira

**Affiliations:** 1 Anesthesiology, Centro Hospitalar Tondela, Viseu, PRT

**Keywords:** respiratory failure, pre-eclampsia, epidural anesthesia, cesarean section, pulmonary edema

## Abstract

Although rare, acute pulmonary edema is the most common cause of death in pregnant women with severe hypertension.

The authors report a case involving a pregnant woman at 36 weeks of gestation with preeclampsia, who exhibited pulmonary edema and required immediate cesarean delivery. The patient had type 1 respiratory failure and needed supplemental oxygenation with a Venturi mask. The cesarean section was performed successfully under epidural anesthesia.

Women with severe preeclampsia and acute pulmonary edema remain a challenge when presented for cesarean delivery, and the best anesthetic option remains controversial.

## Introduction

Acute pulmonary edema is a well-known complication of preeclampsia and contributes to increasing maternal and perinatal morbidity and mortality. It may occur in up to 2.9% of women with preeclampsia and 30% before delivery. It is the most common cause of death in pregnant women with complications of hypertension and a frequent cause of admission to the intensive care unit (ICU) [1].

Because of the physiologic changes that occur during pregnancy, pregnant women are more likely to develop pulmonary edema. In normal pregnancy, pulmonary vascular resistance decreases significantly, and in preeclampsia, pulmonary capillary permeability increases. The combination of these two factors increases the risk of pulmonary edema.

It is crucial to identify preeclamptic women at risk and have a skilled multidisciplinary team to manage these critical situations. The early intervention with hemodynamic optimization and adequate oxygenation leads to a successful outcome [1].

The authors describe a case of a pregnant woman with preeclampsia and pulmonary edema who presented for urgent cesarean delivery (C-section) that was successfully managed with an epidural anesthesia technique.

## Case presentation

A 34-year-old primigravida at 36 weeks of gestation presented to the emergency department with complaints of dyspnea and moderate-intensity nonspecific left-sided chest pain. The pain did not radiate and lacked classical anginal characteristics. She presented a blood pressure (BP) of 142/90 mmHg, heart rate (HR) of 102 beats per minute, respiratory rate (RR) of 30 breaths per minute (bpm), and oxygen saturation of 99% on room air. Her laboratory findings revealed elevated troponin I (240 ng/L; normal values 29-74 ng/L). Blood count and coagulation tests were within normal values. Additionally, there was no evidence of proteinuria, and the urine protein/creatinine (Pr/Cr) ratio was within the expected limits. The patient was evaluated by a cardiologist. An electrocardiogram was performed that did not show any signs of acute coronary syndrome. A transthoracic echocardiogram was performed, revealing mild/moderate mitral regurgitation and mild tricuspid regurgitation. Deep vein thrombosis, pulmonary thromboembolism, and pulmonary edema diagnosis were excluded by ultrasonography and computed tomography angiography. These results and the decrease in biomarkers of myocardial necrosis (repeated nine hours later, troponin I: 70 ng/L) excluded cardiac acute pathology. Dyspnea was associated with fetal position, and the patient was discharged home with an appointment scheduled within five days.

Five days later, the patient maintained dyspnea and presented hypertension (BP 156/93 mmHg) and urine Pr/Cr ratio >0.3. The complete blood cell count, metabolic profile, liver function tests, and coagulation tests were normal. She was admitted to the ward with a diagnosis of preeclampsia. Antenatal corticosteroid therapy for fetal maturation was started with dexamethasone. Two days later, the patient kept hypertension (requiring administration of nifedipine), proteinuria, and dyspnea, and the obstetric team decided to perform an urgent C-section. No fetal distress was reported. Preoperative anesthesiologist evaluation was requested at this time. The patient was dyspneic (while seated at a 90-degree angle) and tachypneic (30 bpm) and found it intolerable to assume a dorsal decubitus position. Physical examination revealed pulmonary rales and edema of the lower limbs and a BP of 143/87 mmHg. Airway evaluation indicated a Mallampati class 1, an inter-incisor distance of approximately 2cm, a thyromental distance of 6cm, and retained cervical mobility. Arterial blood gas analysis revealed type 1 respiratory failure (pH 7.45; pCO2 25 mmHg; pO2 57 mmHg; HCO3- 17.4 mmol/L; SpO2 91%), with PaO2/FiO2 ratio of 247 mmHg on room air. Her laboratory findings remain within normal values. Transthoracic echocardiogram and electrocardiogram did not reveal significant alterations. The diagnosis of severe preeclampsia with pulmonary edema was made. Supplemental oxygenation was initiated with a Venturi mask, and an intravenous (IV) infusion of magnesium sulfate and IV furosemide bolus (total of 40 mg) was administered. The intensive care unit was available if intensive care support was needed.

Upon arrival at the operation room, her vital signs were BP 155/80 mmHg, HR 140 beats per minute, RR 24 bpm, and oxygen saturation of 94% on FiO2 0.28. An additional bolus of furosemide was administered, and a noninvasive ventilation device was available if needed.

An epidural catheter was positioned at the L2-L3 level while the patient was seated. Following the administration of a 60 mg lidocaine 2% dose test, a carefully titrated slow bolus of 0.75% ropivacaine (90 mg) and 50 mcg fentanyl was given. After 25 minutes, a T4-sensitive block was achieved. The angle of the head section of the surgical table was slowly lowered according to patient tolerance, to a minimum of 30 degrees, and a C-section was performed. We opted for restrictive fluid therapy in the perioperative period, and bladder catheterization was obtained to monitor urinary output.

**Figure 1:**
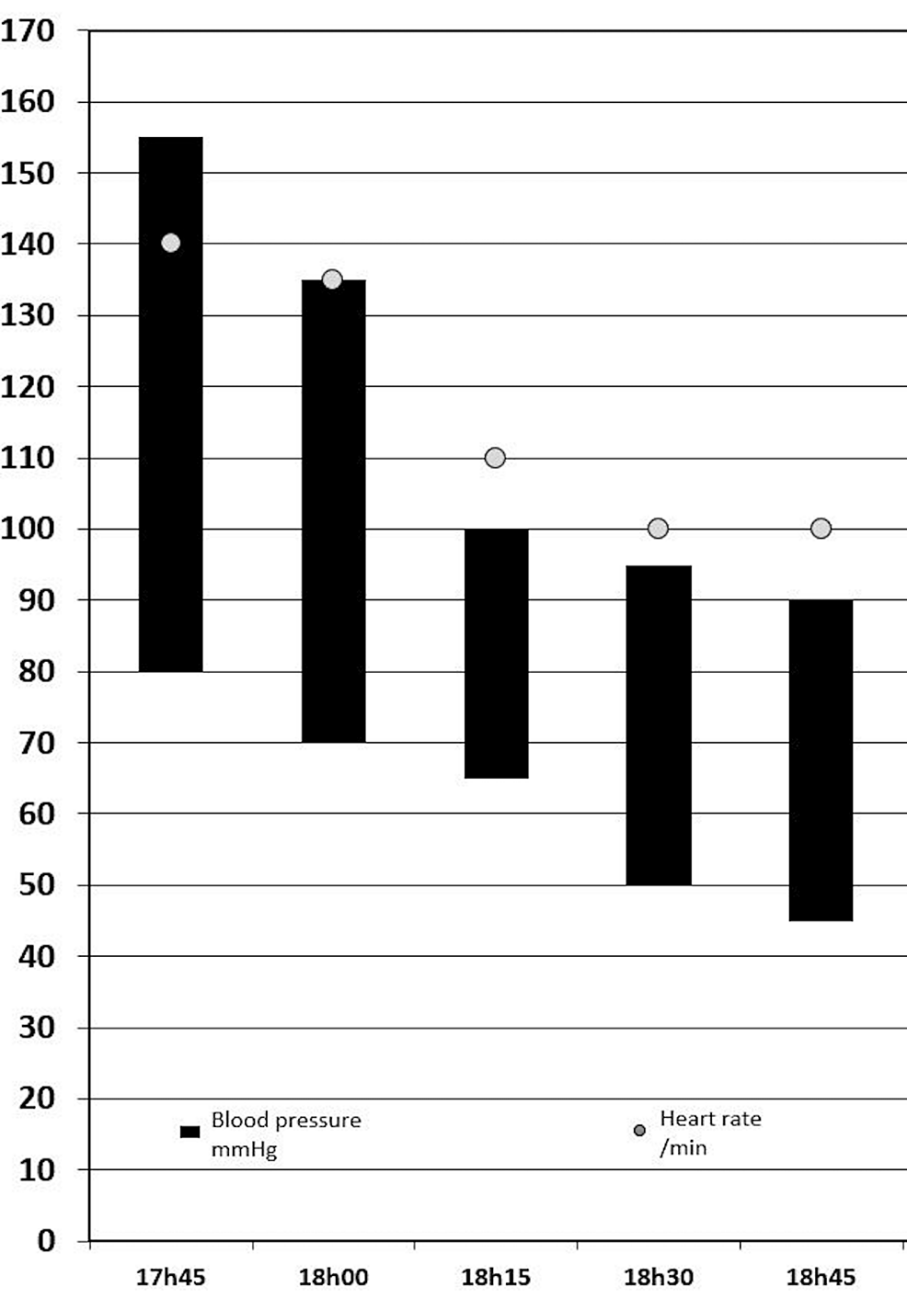
Evolution of blood pressure during surgery.

A female infant was delivered with an Apgar score of 9/10/10. During surgery, oxygen through a Venturi mask on FiO2 0.28 and IV infusion of magnesium sulfate was maintained. The patient remained hemodynamically stable (Figure 1) and reported progressive clinical symptomatic improvement. Clinical and gasometric improvement continued throughout the stay in the postanesthesia care unit (PACU) (FiO2 0.28; pH 7.40; pCO2 31 mmHg; pO2 81 mmHg; HCO3- 19.2 mmol/L; SpO2 95%).

**Figure 2:**
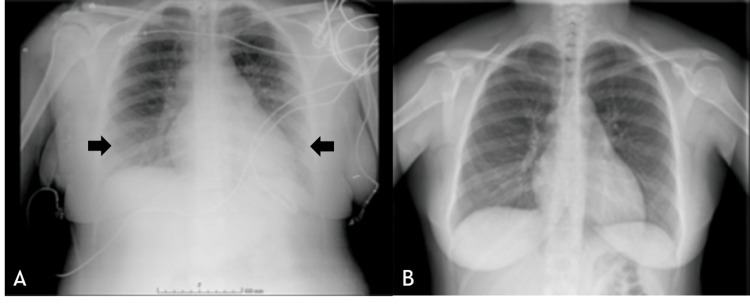
(A) Chest X-ray performed in PACU after delivery, showing marked hilar engorgement and diffuse bilateral infiltrate (black arrows); (B) chest X-ray performed five days after delivery, revealing no alterations. PACU, postanesthesia care unit

Chest X-ray was performed, showing marked hilar engorgement and diffuse bilateral infiltrate (Figure 2A). The patient was transferred to the ward after six hours on PACU maintaining electrocardiography (ECG), noninvasive blood pressure (NIBP), and oxygen saturation monitorization.

The patient continued to receive diuretic therapy with furosemide and magnesium sulfate. Supplemental oxygen was only needed for the next two days. Five days later, both transthoracic echocardiogram and chest X-ray (Figure 2B) revealed no alterations. She was discharged home on the sixth day, with a scheduled cardiology appointment.

## Discussion

Preeclampsia is a multisystemic disorder with unique concerns for the anesthesiologist in the peripartum period. These women are at increased risk of life-threatening events, namely, placental abruption, cerebral hemorrhage, pulmonary edema, acute kidney injury, hepatic failure or rupture, disseminated intravascular coagulation, and progression to eclampsia [2].

Pulmonary edema is a serious complication of severe preeclampsia and may be either cardiogenic or noncardiogenic. Cardiogenic pulmonary edema is due to either impaired left ventricular systolic or diastolic function. Noncardiogenic pulmonary edema can result from multiple factors, such as increased capillary permeability, iatrogenic fluid overload, an imbalance between colloid osmotic pressure and hydrostatic pressure, or a combination of these. IV fluid administration is the major preventable factor [1,3].

The definitive treatment of preeclampsia is delivery. In the case of acute pulmonary edema, the treatment depends, additionally, on the severity of hypoxemia and the underlying disease. The initial treatment involves supplemental oxygen and hemodynamic optimization aimed at reducing left ventricular preload and afterload. This can be achieved through the use of venodilators, diuretics, and positive pressure ventilation [1]. Despite the risk of aspiration in pregnant women, oxygen delivery using noninvasive ventilation with bilevel positive airway pressure (BiPAP) or continuous positive airway pressure (CPAP) should be tried previously for tracheal intubation and mechanical ventilation. The benefit relies on the positive pressure that is applied to the airways, moving fluid from the alveoli into the pulmonary circulation. It also reduces the work of breathing and the need for invasive ventilation [4,5].

The preanesthesia evaluation of these patients should focus on the severity of the disease, airway evaluation, hemodynamic status, coagulation parameters, and fetal status, all of which may change over time. In patients with severe preeclampsia, the choice of anesthetic technique is challenging. Some studies comparing general versus regional anesthesia report a risk-benefit favor for neuraxial blockade in the absence of contraindications [2].

Neuraxial anesthesia has the benefit of maintaining the mother's wakefulness during delivery and minimizing anesthetic exposure to the neonate. It lessens the neuroendocrine response and allows the administration of neuraxial opioids, decreasing postoperative pain. It minimizes the risk of failed intubation, ventilation, and aspiration, as well as the exacerbated hypertensive response to laryngoscopy [2,5]. In this case, beyond the reasons presented before, induction of general anesthesia would probably be associated with severe desaturation. Pregnancy is associated with a reduction of functional residual capacity (FRC), and this falls further with the supine position. The time to desaturation during apnea is halved by the combination of reduced FRC and increased oxygen consumption. In this case, the patient already presented type 1 respiratory failure, so the desaturation time would be even faster.

Typical neuraxial techniques for C-sections include single-shot spinal technique, epidural catheter technique, or combined spinal-epidural (CSE) technique. Spinal anesthesia is an easier technic, with a faster and more reliable onset of surgical anesthesia but may be associated with abrupt hemodynamic alterations. Epidural anesthesia permits a slow onset of sympathectomy, a titrated dosing, and the use of postoperative analgesia. However, there is no scientific evidence of a technique’s advantage over others [2]. In this case, epidural anesthesia was selected over a spinal approach to allow careful drug titration and better hemodynamic stability.

General anesthesia is typically used when neuraxial anesthesia is contraindicated or failed, or in emergencies because of its rapid and predictable effect [2,5]. 

In the literature, there are a few reports of anesthetic management of acute pulmonary edema during C-section. Pancholi et al. described a case of pulmonary edema during an emergency C-section, under spinal anesthesia, that was successfully managed with noninvasive ventilation [5]. Tayde et al. reported a case of preeclampsia with pulmonary edema that needed general anesthesia due to critical hypoxemia and hemodynamic instability [1].

In this case, the patient was diagnosed with severe preeclampsia with acute pulmonary edema. The choice of epidural anesthesia technique was determined according to the clinical, anesthetic, and obstetrical circumstances. A progressive titrated bolus of local anesthetics and opioids allowed an adequate surgical block, maintaining hemodynamic stability and clinical improvement of dyspnea. Epidural anesthesia, by promoting venodilation, helps reduce preload and subsequently decreases the pressure within the pulmonary vasculature. This improves cardiac output and enhances oxygenation. At the same time, diuretic therapy targets excess fluid retention by increasing urine output, thereby reducing both intravascular volume and pulmonary congestion. The combination of these two interventions leads to a reduction in pulmonary edema and an overall enhancement in the patient's respiratory status, probably justifying the noticeable clinical and gasometric improvement.

Although administering an IV fluid bolus during regional anesthesia is a routine practice to reduce hypotension, fluid therapy for patients with preeclampsia undergoing a C-section remains a subject of controversy, and there is a paucity of evidence-based guidelines concerning IV fluid administration [2,3]. The decision needs to be carefully weighed, considering the risk of iatrogenic pulmonary edema or exacerbation of an existing condition. In the reported case, restrictive fluid therapy was adopted.

Multimodal treatment and epidural anesthesia, ensuring optimal oxygenation and stable hemodynamics with restrictive fluid therapy, seemed to be justifiable in this case.

## Conclusions

Preeclampsia is a perioperative medical challenge. In this clinical scenario, the decision to perform epidural anesthesia represented a thoughtful and strategic choice that allowed to promote hemodynamic stability, optimize cardiac function, and consequently improve oxygenation. It highlights the importance of individualized care, showing how the gradual onset, sympathetic-sparing effects, and meticulous dose adjustment of epidural anesthesia can mitigate the risks associated with pulmonary congestion, cardiac strain, and compromised respiratory function.

Ensuring the safety and care of these patients necessitates a multidisciplinary team approach that involves the patient and emphasizes clear and open communication.
